# Opportunities for Innovation in Genetic Transformation of Forest Trees

**DOI:** 10.3389/fpls.2018.01443

**Published:** 2018-10-02

**Authors:** Michael Nagle, Annabelle Déjardin, Gilles Pilate, Steven H. Strauss

**Affiliations:** ^1^Forest Ecosystems and Society, Molecular and Cellular Biology, Oregon State University, Corvallis, OR, United States; ^2^BioForA, INRA, ONF, , Orléans, France

**Keywords:** transformation, regeneration, *WUSCHEL*, *BABY BOOM*, *Populus*, organogenesis, embryogenesis, Agrobacterium

## Abstract

The incorporation of DNA into plant genomes followed by regeneration of non-chimeric stable plants (transformation) remains a major challenge for most plant species. Forest trees are particularly difficult as a result of their biochemistry, aging, desire for clonal fidelity, delayed reproduction, and high diversity. We review two complementary approaches to transformation that appear to hold promise for forest trees.

## Summary

Developmental genes (DG) may be useful tools for promoting transformation. DGs, which can act through a wide variety of developmental mechanisms to promote regeneration of transgenic cells, have been widely employed in model plants to promote embryogenesis and in some cases organogenesis. Following initial experimental demonstration in dicots, the DGs *WUSCHEL* and/or *BABY BOOM* have formed the basis of a high efficiency method for a variety of monocot genotypes and species. However, in dicots the utility of these genes as the basis of a robust transformation system has not been demonstrated. Many additional DGs appear capable of promoting regeneration that have not been systematically explored as transformation tools.

Because *in vitro* plant transformation systems are costly and must be customized for each new genotype and species, *in vivo* approaches to transformation hold much appeal. It is possible to produce stable transgenic plants by agro-inoculation of seeds or vegetative/floral buds, but as yet these approaches have not been used routinely in any plant species except for the Arabidopsis floral dip. We will discuss how the Arabidopsis system, and other *in planta* techniques, may be tailored for forest trees, taking into account variations in biology of different taxa.

## Developmental Genes as Tools for Transformation Improvement

**FIGURE 1:**
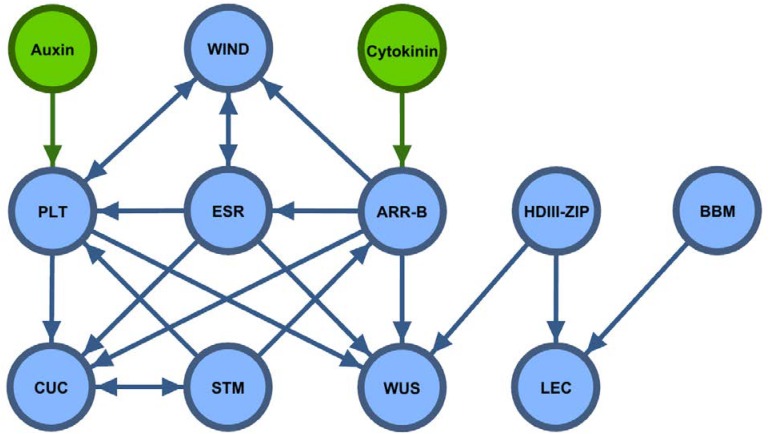
Portion of genetic regulatory network with demonstrated ability to enhance regeneration via overexpression. Green nodes represent phytohormones and green arrows indicate transactivation that results from upstream hormone-triggered cascades (not shown). Auxin leads to PLT upregulation through a relatively well-characterized cascade (Horstman et al., 2014), as do cytokinins for B-type ARRs (Xie et al., 2018). Blue nodes represent genes which have been shown to enhance regeneration when overexpressed, and blue arrows indicate direct targets of their transactivation activity (detailed in **Supplementary Table 1**).

When overexpressed, transcriptional or epigenetic regulators of embryo and meristem development (referred to as developmental genes, DGs) have been shown to confer improvements to *in vitro* regenerability. Recent molecular evidence places these genes within a genetic regulatory network, connected by cascades and feedback loops of transactivation. Knowledge of these interactions, detailed in **Supplementary Table 1** and **Figure 1**, can inform selection of individual genes and combinations of genes that may be most effective for improving regeneration. In this mini-review, however, we do not consider the many genes which may enhance regeneration via epigenetic mechanisms, or by affecting the rate of gene transfer or incorporation of DNA into the genome.

### Candidate Genes and Their Modes of Action

The first gain-of-function mutation for a transcriptional regulator of shoot induction was observed in 1941. Bryan and Sass discovered a heritable trait in maize that causes leaves to develop ectopic shoot meristems, or “knots” (Bryan and Sass, 1941). Transposon tagging later revealed the responsible locus to be *Knotted-1*, the first homeodomain transcription factor identified in plants (Hake et al., 1989). *Knotted-1* was found to be overexpressed in mutants as a result of a transposon insertion (Smith et al., 1992), and overexpression of maize *Knotted-1* in tobacco and Arabidopsis was also reported to trigger development of ectopic shoot meristems (Sinha et al., 1993; Lincoln et al., 1994).

A loss-of-function mutation of *SHOOT MERISTEMLESS* (*STM*), an Arabidopsis homolog of *Knotted-1*, was reported to lead to premature termination of shoot meristems and a phenotype of twig-like plants lacking lateral shoots (Long et al., 1996). Transcription of *STM* is regulated in part by a positive feedback loop between *STM* and the transactivator *CUP-SHAPED COTYLEDON 1* (*CUC1*) (**Supplementary Table 1** and **Figure 1**; Takada et al., 2001; Spinelli et al., 2011). Similarly to STM, overexpression of CUC1 enhances regeneration through embryogenic and organogenic pathways (Takada et al., 2001; Hibara et al., 2003). Overexpression of *CUC1/2* in Arabidopsis led to large increases in numbers of shoot from calli, while knockout had the opposite effect (Daimon et al., 2003). Directly upstream of *CUC1* is *LEAFY COTYLEDON1 (LEC1)*, which was first identified as a regulator of embryogenesis when overexpression induced embryos to develop on leaves of Arabidopsis (Lotan et al., 1998).

A mutant screen for shoot meristem defects led to the discovery of *WUSCHEL (WUS)* loss-of-function mutants, which had similar phenotypes to *stm* mutants (Laux et al., 1996). *WUS* is expressed in the organizing center adjacent to meristematic stem cells (Mayer et al., 1998), then trafficked into the central zone of the stem cell niche (Yadav et al., 2010; Daum et al., 2014), where it is required for maintenance of stem cell identity. Contrarily, *STM* is expressed and active in the peripheral zone (PZ) (Williams et al., 2005; Gordon et al., 2007). In Arabidopsis, overexpression of *WUS* was reported to initiate ectopic organogenesis *in vivo*, although differentiation into organs was incomplete unless *STM* was also overexpressed (Gallois et al., 2002). *WUS* was rediscovered in a T-DNA activation mutagenesis screen for gain-of-function mutations conferring cytokinin-independent potential for induced embryogenesis *in vitro* (Zuo et al., 2002).

Downstream of *WUS* and *STM* are overlapping sets of diverse genes which balance differentiation and dedifferentiation to promote progressive development of meristems. These genes include members of the SE-associated receptor kinase (SERK) family, as well as enzymes for hormone biosynthesis, cell cycle regulators, and numerous transcriptional regulators, some of which function not only downstream, but upstream of *WUS*/*STM* (Spinelli et al., 2011; Balkunde et al., 2017; Ikeuchi et al., 2018; Scofield et al., 2018).

*WUS* transcription is activated by a complex of factors including B-type *ARABIDOPSIS RESPONSE REGULATOR (ARR)* and HDIII-ZIP family proteins (**Supplementary Table 1** and **Figure 1**). In separate experiments, overexpression of *ARR2* and *ARR12* led to roughly twofold increases in number of shoots from callus *in vitro* (Dai et al., 2017; Zhang et al., 2017). Contrary to the roles of B-type *ARR* genes, overexpression of the A-type led to strong suppression of *in vitro* organogenic capacity (Osakabe et al., 2002; Buechel et al., 2010).

The combined knockout of several HDIII-ZIP factors in a *wus* background rescued SAM development, indicating that these factors may simultaneously promote and inhibit stem cell differentiation via pathways both dependent and independent of *WUSCHEL* (Lee and Clark, 2015). Orthologs of these TFs found in *Populus trichocarpa* are expressed in the SAM, and overexpression promotes stem cell proliferation and inhibits development of shoot primordia into mature organs (Du et al., 2011; Robischon et al., 2011; Zhu et al., 2013).

*ENHANCER OF SHOOT REGENERATION 1/2* acts directly upstream of *WUS* and indirectly upstream of *STM* (**Supplementary Table 1** and **Figure 1**) and numerous other poorly known genes (Chandler et al., 2007; Ikeuchi et al., 2018). *ESR1* overexpression conferred cytokinin-independent competence for regeneration, although constitutive overexpression inhibited differentiation of SAMs. Recovery of transgenic plants was enabled via deactivation of chemoinducible *ESR1* after shoot primordial development (Banno et al., 2001). *ESR2* is transactivated by *ESR1* and shares many downstream targets (Ikeuchi et al., 2018); overexpression of either leads to remarkable improvement in shoot regeneration capacity in Arabidopsis (Ikeda et al., 2006; Mase et al., 2007). Unlike *ESR1* (Banno et al., 2001), overexpression of the upstream gene *WOUND-INDUCED DEDIFFERENTIATION 1 (WIND1)* enhances formation of callus in addition to shoots (Iwase et al., 2016); however, callus develops into shoots upon transfer of chemoinducible *wind1* mutants to media without inducer (Iwase et al., 2011). Several WIND homologs are known to transcriptionally activate ESR1 (**Supplementary Table 1**).

*PLETHORA (PLT) 3/5/7* are responsible for direct transactivation of WUS and indirect transactivation of both WUS and STM via *CUP-SHAPED COTYLEDON (CUC) 1/2* (**Supplementary Table 1** and **Figure 1**). In Arabidopsis, overexpression of *PLT5/7* enables cytokinin-independent shoot regeneration, although at a very low rate (Kareem et al., 2015).

*BABY BOOM (BBM)* overexpression confers the ability for cytokinin-independent *in vitro* somatic embryogenesis in Arabidopsis (Boutilier et al., 2002). In contrast, expression of a *BBM* homolog in tobacco enhanced regeneration via shoot organogenesis without affecting embryogenesis (Srinivasan et al., 2007, 2011).

### Effectiveness of Developmental Genes in Non-model Species

*WUS* and related genes have been found to be effective at promoting regeneration in crop and forest species. Overexpression of the rice homolog of *WUS* in seedlings was found to cause *de novo* organogenesis of shoots in rice (Kamiya et al., 2003). *Populus tomentosa* transformed to overexpress any of four *WUSCHEL* or *WUSCHEL-ASSOCIATED HOMEOBOX* orthologs showed increased adventitious rooting (Liu et al., 2014; Li et al., 2018). Overexpression of *AtWUS* led to increased embryo and callus formation *in vitro* in coffee (Arroyo-Herrera et al., 2008), increased embryo formation in cotton (Bouchabké-Coussa et al., 2013), and enables *in vitro* ectopic embryogenesis for the otherwise completely recalcitrant *Capsicum chinense* (Solís-Ramos et al., 2009). Overexpression of homologs of *WUS*, or *WUS* in combination with *BABY BOOM (BBM)*, enhances *in vitro* transformation and shoot regeneration in a variety of monocots, including rice, sorghum, and maize. Several completely recalcitrant maize inbred lines became responsive to transformation and regeneration when overexpressing either *WUS* or *BBM* homologs (Lowe et al., 2016; Mookkan et al., 2017).

*STM* and related genes are also active in crop and forest species. Expression of *STM/Knotted-1* orthologs from apple or maize enhanced shoot regeneration from leaf explants in the absence of exogenous cytokinins in tobacco, though were not effective in plum under the conditions studied (Srinivasan et al., 2011). In citrus, expression of maize *Knotted-1* enhanced *in vitro* regeneration after transformation, with rates varying widely among varieties (Hu et al., 2016). In the gymnosperm forest tree *Picea abies* (Norway spruce), overexpression of a *KNOTTED-1* ortholog similarly promoted *in vitro* somatic embryogenesis (Belmonte et al., 2007).

Other DGs can also stimulate regeneration in non-model species. Overexpression of maize *LEC* in wheat and maize enabled efficient transformation without the use of selectable markers (Lowe et al., 2002). *AtLEC1* overexpression in white spruce, however, had no effect on somatic embryogenesis (Klimaszewska et al., 2010). Overexpression of an *ESR1* ortholog led to a doubling of shoot regeneration during transformation in hybrid poplar (Yordanov et al., 2014). Overexpression of a *BBM* ortholog in *Capsicum annuum* (sweet pepper) enabled *in vitro* somatic embryogenesis and efficient transformation of a genotype which was previously recalcitrant (Heidmann et al., 2011). In *P. tomentosa*, expression of a *Brassica* homolog of *BBM* led calli to develop somatic embryos, which is otherwise rarely seen with poplar regeneration systems (Deng et al., 2009).

### Strategies for Using Developmental Genes for Transformation Improvement

When overexpressed during vegetative development, *BBM*, *WUS*, *LEC1* and other genes can promote various regeneration pathways, but then unsurprisingly lead to defects in further development, such as disorganized shoot and floral meristems (Gallois et al., 2002), infertility, and shoot necrosis (Lowe et al., 2016). Clearly, to be useful their expression must be carefully controlled in strength and timing. Three main options exist for targeted expression of transgenes: induced expression using external stimuli such as chemical or physical inducers (e.g., heat or drought); controlled excision of the genes from the genome using similar inducer options; and use of promoters whose innate expression pattern closely resembles that of native meristematic genes, thus will have much attenuated expression after meristems or embryos are initiated. More complex options include gene editing to engineer the promoters of native DGs to be inducible, or to add miRNA-resistance mutations into the genes’ transcribed regions, thus achieving some level of derepression without affecting genomic context and other local regulatory cues (Zhang et al., 2017). Heat-shock, drought-stress, and meristematic promoters have been shown to be effective for driving Cre excision of *BBM* and *WUS* orthologs in monocots (Lowe et al., 2016, 2018; Mookkan et al., 2017), and a heat-shock promoter was used to drive Flp-*FRT* excision of *BBM* after *BBM* induced somatic embryogenesis in *P. tomentosa* (Deng et al., 2009). Clearly many options exist, but the choice of the most effective DGs or combinations thereof, as well as gene expression/removal options, need to be explored in parallel for specific crop and forest tree species.

*In vitro* culture and selection conditions will strongly affect the value of DGs for promoting regeneration. For example, plant hormones added to culture media can mask or help to amplify DG effects, depending on the specific taxa, tissues, and hormone species and concentrations, and duration of treatments (Hill and Schaller, 2013; Irshad et al., 2018; Kumar et al., 2018). With highly effective regeneration, use of selective agents such as antibiotics can be reduced or eliminated entirely, greatly reducing physiological stresses that retard regeneration (Mookkan et al., 2017; Lowe et al., 2018). The optimal combinations, and their impacts on transformation rate as well as chimerism in the resulting plants, can only be determined by trial and error with specific taxa, and may need to be customized to specific genotypes in highly genetically variable and recalcitrant species such as most forest trees.

Future improvements to the system are likely to include growing use of multiple DGs, in part to complement and balance their differing activities, and to make DG “reagents” effective across a wide range of taxa. For example, strong overexpression of *WUS* or upstream *ESR1* without sufficient balancing activity of the *STM* pathway in the PZ has reportedly led to failure of PZ cells to differentiate (Banno et al., 2001; Ikeda et al., 2006), and to necrosis of shoot primordia (Lowe et al., 2016). Studies of gene combinations have to date received little attention, but may lead to major improvements in transformation efficiency.

## Toward the Development of *in planta* Transformation Methods in Forest Trees

A major limitation to genetic transformation is the need to develop *in vitro* propagation and regeneration systems, which for many plant species are very time consuming and require a high level of technical expertise. Moreover, it must be customized for each new genotype and species, with many remaining recalcitrant to regeneration and/or transformation. As a result, methods that bypass the need for *in vitro* systems are highly desirable.

**FIGURE 2:**
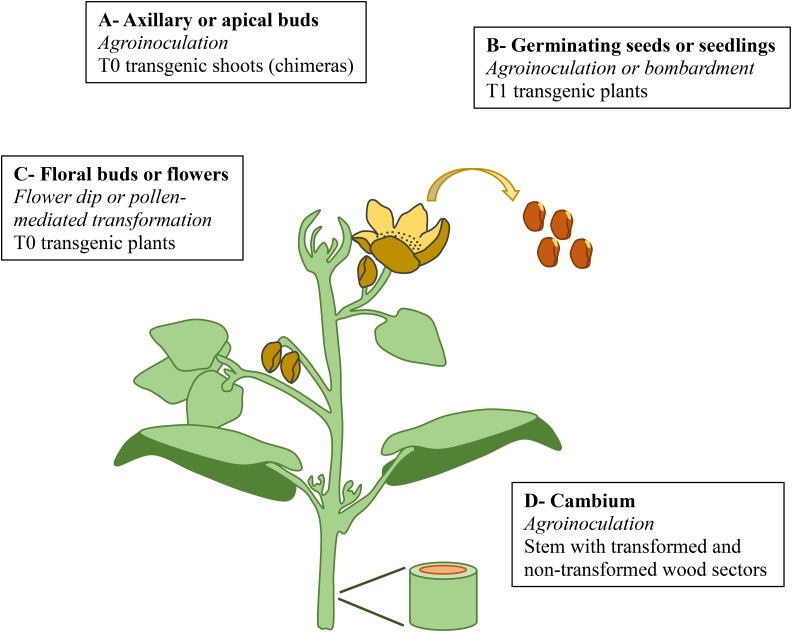
Virtual plant showing the diverse organs/meristems targeted by *in planta* transformation. **(A)** Primary meristems in vegetative buds leading to the production of transgenic shoots after agro-inoculation. **(B)** Primary meristems in germinating seeds or seedlings leading to T0 chimeric plants and T1 transgenic plants. **(C)** Reproductive cells in floral buds or flowers leading to T0 transgenic plants. **(D)** Cambium secondary meristems leading to transformed wood sectors.

*In planta* transformation techniques take advantage of natural biological processes to produce and regenerate transgenic plants (**Figure 2**), and are thus in theory applicable to a large panel of genotypes and species. The target tissues are diverse, and can include secondary meristems. The induced somatic sector analysis (ISSA) approach is an example of what can be achieved in various tree species (*Pinus*, *Eucalyptus*, *Populus*, Spokevicius et al., 2005; Van Beveren et al., 2006). A “cambial window” is cut with a sharp razor blade through the bark to get access to the cambial/young xylem tissues, which are then inoculated with an *Agrobacterium tumefaciens* solution. After wound closure and cambium reestablishment, the transformed cells divide and differentiate, producing somatic sectors of transformed cells. Within a few months, and without any *in vitro* steps or complex manipulations, it is possible to analyze transgenes and promoters directly in the woody stem tissues of trees by comparing transformed sectors with adjacent non-transformed ones (Hussey et al., 2011; Creux et al., 2013; Baldacci-Cresp et al., 2015). ISSA has a great potential to study cell fate and pattern formation during secondary growth and xylogenesis, thanks to the development of microscope-derived techniques like Raman or ATR-FTIR, which can give spectroscopic information at the cellular or cell wall level. Although very useful for research, ISSA can, however, not be used to regenerate transgenic plants.

To this end, other tissues can be targeted for transformation, including vegetative meristems protected in axillary or apical buds, as was investigated in sugarcane (Mayavan et al., 2015), *Populus* (Yang et al., 2010), and grapevine (Fujita et al., 2009). *A. tumefaciens* was the DNA vector and reached the meristems after mechanical wounding, possibly complemented by sonication or vacuum infiltration (Mayavan et al., 2015). This approach required an efficient adventitious rooting system to regenerate plants from transgenic shoots excised from mother plants. However, repeated rounds of selection generally failed to avoid chimeric transgenic shoots.

To avoid chimeras, some protocols have been developed on germinating seeds or seedlings, with the goal of reaching apical meristems as early as possible in plant development. Indeed, these meristems will ultimately give rise to reproductive meristems that may produce transformed germ cells. Several attempts were at least partly successful in producing transgenic T1 plants from T0 transformed chimeric embryos or seedlings using *A. tumefaciens* (Lin et al., 2009; Shah et al., 2015; Ahmed et al., 2018) or particle bombardment (Hamada et al., 2017). However, this method would be difficult to apply in forest trees as the elimination of chimeras usually requires sexual reproduction to the T1 generation, requiring a long wait for flowering.

In order to speed up the process, while ensuring the generation of non-chimeric plants, some authors targeted reproductive meristems before flowering and fertilization. The rationale was to transform future germ cells. Arabidopsis flowers were successfully transformed by *A. tumefaciens* vacuum infiltration (Bechtold et al., 1993); Clough and Bent (1998) further improved this method, and found that dipping of flowers was efficient enough as long as a surfactant was also used. Even though the overall efficiency was less than 1%, the method remained viable thanks to the high fertility and small size of Arabidopsis, enabling hundreds to thousands of germinating seeds to be efficiently screened using selectable markers. This method was developed with limited success in other species (*Raphanus sativus*, Curtis and Nam, 2001; *Brassica napus*, Qing et al., 2000; *Medicago truncatula*, Trieu et al., 2000). An alternative method is to target pollen grains using methods such as sonication to penetrate pollen apertures in a DNA-containing sucrose solution. Southern analyses demonstrated the successful transfer of transgenes to progenies by pollination in sorghum (Wang et al., 2007), *Brassica juncea* (Wang et al., 2008), and maize (Yang et al., 2017). Pre-treatment of pollen grains by aeration (e.g., 20 min at 4°C) increased pollen viability, mitigating a common adverse effect of sonication (Yang et al., 2017). Zhao et al. (2017) have recently investigated an innovative method by which DNA was delivered to pollen via magnetic nanoparticles; stable transformants in cotton, pepper, and pumpkin were generated. Pollen-mediated transformation could be easily tested for forest trees as pollination for controlled crosses or seed production is very common as part of conventional breeding. However, to be feasible and conform to most regulatory requirements for containment of transgenic pollen, such crosses would need to be carried out on detached or grafted floral branches in greenhouses, which is possible only for a limited number of forest tree species.

Unfortunately, most of the procedures described above required a high level of technical expertise, as they were hardly transferred to other laboratories. The only exception is Arabidopsis floral dip, which has been used in numerous laboratories worldwide. Could *in planta* techniques such as this be tailored for forest trees? Several convergent studies have shown that the ovules, not the pollen, are the direct targets for transformation through floral dip. It has been shown that manual outcrossing experiments produced transgenic progenies only when *A. tumefaciens* was applied on pollen recipient plants, not on pollen donor plants (Ye et al., 1999; Desfeux et al., 2000). Bechtold et al. (2000) elegantly reached the same conclusion using a genetic approach.

Floral dip has been shown to give rise to transgenic seedlings with genetically independent insertions that are typically hemizygous (carrying the T-DNA at only one allele of a given locus) (Bechtold et al., 1993). The transformation rate is very dependent on the flower developmental stage (Desfeux et al., 2000), with the optimal stage being when the gynoecium is still open (Irepan Reyes-Olalde et al., 2013), thus allowing agrobacteria to penetrate and transform the ovule primordia.The CRABS-CLAW mutant, which maintains an open gynoecium, gives a sixfold enhanced rate of transformation (Desfeux et al., 2000). Therefore, access by Agrobacterium to the locule of the ovule appears to be critical for the transformation. Trees are perennial species, where flower initiation takes place the year before flowering, thus injection of the Agrobacterium solution into female floral buds before flowering at a stage where the gynoecium is still open needs to occur weeks to months before seed release. However, trees also produce very large number of seeds, thus the *in planta* approach, if it can be optimized and applied to many buds at the right times, together with an efficient selection system for germinating seeds, may be realistic option for some tree species.

## Conclusion

In summary, both DG and *in planta* approaches to transformation hold promise to solve major problems in plant and tree transformation. DGs appear to hold most promise where a basic *in vitro* regeneration system is in place, thus might benefit from a large elevation of transformation efficiency using an established transformation pathway. It has to date been most effective in species with embryogenic rather than organogenic regeneration systems. *In planta* systems hold most promise where *in vitro* approaches are extremely difficult or impossible, thus alternate pathways are required. It will also be most easily pursued as part of a large-scale breeding program, enabling large numbers of floral buds to be treated and seedling populations screened for transformation and chimerism. *In planta* transformation of cambium and axillary/apical buds has been successful, but is prone to chimerism; research on germline transformation (i.e., transformation of mother cells within floral buds and embryos within seeds) may help to reduce this problem. *In planta* and DG overexpression approaches to efficient transformation might not be mutually exclusive; research is warranted to elucidate the potential for DGs to enhance *in planta* systems. Given the importance of regeneration as a bottleneck to transformation and gene editing of forest trees in research and application (Chang et al., 2018), acceleration of research using both approaches is warranted.

## Author Contributions

MN and AD drafted the sections “Developmental Genes as Tools for Transformation Improvement” and “Toward the Development of *in planta* Transformation Methods in Forest Trees,” respectively, after extensive consultation with SS and GP. SS and GP revised the drafted text and prepared initial drafts of the sections “Introduction” and “Conclusion.”

## Conflict of Interest Statement

The authors declare that the research was conducted in the absence of any commercial or financial relationships that could be construed as a potential conflict of interest.
