# Corrigendum: Opportunities for Innovation in Genetic Transformation of Forest Trees

**DOI:** 10.3389/fpls.2018.01729

**Published:** 2018-11-28

**Authors:** Michael Nagle, Annabelle Déjardin, Gilles Pilate, Steven H. Strauss

**Affiliations:** ^1^Forest Ecosystems and Society, Molecular and Cellular Biology, Oregon State University, Corvallis, OR, United States; ^2^BioForA, INRA, ONF, Orléans, France

**Keywords:** transformation, regeneration, *WUSCHEL*, *BABY BOOM*, *Populus*, organogenesis, embryogenesis, Agrobacterium

In the published article, there was an error in affiliations 1 and 2. The affiliations were switched for authors Annabelle Déjardin and Steven H. Strauss, who are respectively affiliated with BioForA, INRA, ONF, Orléans, France and Oregon State University, Corvallis, OR, United States. The authors apologize for this error and state that this does not change the scientific conclusions of the article in any way. The original article has been updated.

## Conflict of interest statement

The authors declare that the research was conducted in the absence of any commercial or financial relationships that could be construed as a potential conflict of interest.

