# Supplementary material for: Opportunities for Innovation in Genetic Transformation of Forest Trees
Source: Front Plant Sci. 2018 Oct 2;9:1443. doi: 10.3389/fpls.2018.01443 (PMC6176273; doi:10.3389/fpls.2018.01443)
Supplement: Supplementary file 1 [file Table_1.docx]

**Supplementary Table 1.** Evidence for interactions between genes with demonstrated potential to improve *in vitro* regenerability when overexpressed.

*Table 1A. Transcriptional regulation*

| Transactivator | Target | Evidence | | Reference | |
| --- | --- | --- | --- | --- | --- |
| B-type ARR ARR1 | CUC1 | Yeast one-hybrid | | Ikeuchi et al., 2018 | |
| B-type ARR ARR1 | ESR2 | Yeast one-hybrid | | Ikeuchi et al., 2018 | |
| B-type ARR ARR1 | WIND3 | Yeast one-hybrid | | Ikeuchi et al., 2018 | |
| B-type ARRs ARR1, ARR10 and ARR12 | WUS | Yeast one-hybrid, ChIP-qPCR, EMSA | | Meng et al., 2017 | |
| B-type ARRs ARR1 and ARR2 | WUS | ChIP-qPCR, EMSA, *in situ* hybridization of *WUS* transcripts in mutants with dominant negative variant of ARR2, luciferase-based assays of pWUS activity | | Zhang et al., 2017 | |
| B-type ARR ARR12 | WUS | ChIP-qPCR, upregulation of pWUS::LUC reporter in p35S:ARR12 mutants | | Dai et al., 2017 | |
| B-type ARR ARR20 | WUS | Yeast one-hybrid | | Ikeuchi et al., 2018 | |
| BBM | LEC1 and LEC2 | qRT-PCR of LEC1/2 in somatic embryos with induction of BBM-GR (during cycloheximide treatment), ChIP-seq | | Horstman et al., 2017 | |
| CUC1 | STM | pSTM::GUS expression in 35S:CUC1 plants, RT-PCR and *in situ* hybridization in CUC1 overexpression and loss-of-function lines | | Hibara et al., 2003; Takada et al., 2001 | |
| HDIII-ZIP TF REVOLUTA | WUS | ChIP-qPCR | | Zhang et al., 2017 | |
| ESR1 | ESR2, WUS, PLT3 | Yeast one-hybrid | | Ikeuchi et al., 2018 | |
| ESR2 | CUC1 | CUC1 expression (detected by microhybridization) in ESR2-RNAi and ESR2-OE lines, and in ESR2-ER lines with induction during cyclohexamide treatment | | Ikeda et al., 2006 | |
| HDIII-ZIP TF PHB | LEC2 | qRT-PCR of LEC2 in *phb* mutants, ChiP-qPCR | | Tang et al., 2012 | |
| STM | ARR12 | Yeast one-hybrid | | Ikeuchi et al., 2018 | |
| STM | CUC1, PLT3/7 | Upregulation of targets when STM-GR induced during cyclohexamide treatment | | Scofield et al., 2018 | |
| STM | CUC1/2/3 | CUC1/2/3: RT-PCR in STM-GR lines,  CUC1: induction of STM-GR in pCUC1::GUS double mutant, EMSA | | Spinelli et al., 2011, Scofield et al., 2018 | |
| PLT3 | WIND3, WUS | Yeast one-hybrid | | Ikeuchi et al., 2018 | |
| PLT3/5/7 | CUC1/2 | qRT-PCR of CUC1/2 in *plt3/5/7* mutants, and in PLT5-GR lines induced during cycloheximide treatment | | Kareem et al., 2015 | |
| WIND1 | ESR1 | ChIP, EMSA, increasing luciferase signal in pESR1::LUC explants upon particle bombardment with WIND1 overexpression constructs | | Iwase et al., 2016 | |
| WIND2 | WIND3 | Yeast one-hybrid | | Ikeuchi et al., 2018 | |
| ESR1 | WIND3 | Yeast one-hybrid | | Ikeuchi et al., 2018 | |
| WIND4 | PLT3 | Yeast one-hybrid | | Ikeuchi et al., 2018 | |
| *Table 1B. Other associations* | |  |  | |  |
| Interaction |  |  | Evidence | | Reference |
| protein-protein interaction between HDIII-ZIP TFs PHB/PHV/REV with ARR1/2 | |  | Yeast two-hybrid, pull-down, CoIP, bimolecular luminesence complementation | | Zhang et al., 2017 |
| protein-protein interaction between ESR1/2 and HDIII-ZIP TFs PHV, PHB, REV, COR, and ATHB8 | |  | Yeast two-hybrid, CoIP, bimolecular luminesence complementation | | Chandler et al., 2007 |
| Methylation of pWUS by MET1 and CMT3 | |  | bisulfite sequencing of pWUS and qRT-PCR of WUS in *met1*, *cmt3* mutants | | Li et al., 2011; Shemer et al., 2015 |
| histone modifications by KYP, JMJ14 and HAC1 downregulating WUS expression | |  | qRT-PCR of WUS in *kyp, jmj14, hac1* mutants | | Li et al., 2011 |
| Methylation of pWUS by DRM1/2 and CMT3 | |  | RT-PCR of WUS in triple mutants of *drm1, drm2* and *cmt3* | | Shemer et al., 2015 |

**References**

Chandler, J.W., Cole, M., Flier, A., Grewe, B., and Werr, W. (2007). The AP2 transcription factors DORNROSCHEN and DORNROSCHEN-LIKE redundantly control Arabidopsis embryo patterning via interaction with PHAVOLUTA. Development *134*, 1653–1662. doi: 10.1242/dev.001016

Dai, X., Liu, Z., Qiao, M., Li, J., Li, S., and Xiang, F. (2017). ARR12 promotes de novo shoot regeneration in Arabidopsis thaliana via activation of WUSCHEL expression. J Integr. Plant Biol. *59*, 747–758. doi:10.1111/jipb.12567

Hibara, K., Takada, S., and Tasaka, M. (2003). CUC1 gene activates the expression of SAM-related genes to induce adventitious shoot formation. Plant J. *36*, 687-696. doi:10.1046/j.1365-313X.2003.01911.x

Horstman, A., Li, M., Heidmann, I., Weemen, M., Chen, B., Muiño, J.M., Angenent, G.C., and Boutilier, K. (2017). The BABY BOOM transcription factor activates the LEC1-ABI3-FUS3-LEC2 network to induce somatic embryogenesis. Plant Physiol. pp.00232.2017. doi: 10.1104/pp.17.00232

Ikeda, Y., Banno, H., Niu, Q.W., Howell, S.H., and Chua, N.H. (2006). The ENHANCER of SHOOT REGENERATION 2 gene in Arabidopsis regulates CUP-SHAPED COTYLEDON 1 at the transcriptional level and controls cotyledon development. Plant Cell Physiol. *47*, 1443–1456. doi:10.1093/pcp/pcl023

Ikeuchi, M., Shibata, M., Rymen, B., Iwase, A., Baagman, A.M., Watt, L., Coleman, D., Favero, D.S., Takahashi, T., Ahnert, S.E., et al. (2018). A gene regulatory network for cellular reprogramming in plant regeneration. Plant Cell Physiol. *59*, 770–782. doi:10.1093/pcp/pcy013

Iwase, A., Harashima, H., Ikeuchi, M., Rymen, B., Ohnuma, M., Komaki, S., Morohashi, K., Kurata, T., Nakata, M., Ohme-Takagi, M., et al. (2016). WIND1 promotes shoot regeneration through transcriptional activation of ENHANCER OF SHOOT REGENERATION1 in Arabidopsis. Plant Cell *29*, 54-69. doi:10.1105/tpc.16.00623

Kareem, A., Durgaprasad, K., Sugimoto, K., Du, Y., Pulianmackal, A.J., Trivedi, Z.B., Abhayadev, P.V., Pinon, V., Meyerowitz, E.M., Scheres, B., et al. (2015). PLETHORA genes control regeneration by a two-step mechanism. Curr. Biol. *25*, 1017–1030. doi:10.1016/j.cub.2015.02.022

Li, W., Liu, H., Cheng, Z.J., Su, Y.H., Han, H.N., Zhang, Y., and Zhang, X.S. (2011). Dna methylation and histone modifications regulate de novo shoot regeneration in arabidopsis by modulating wuschel expression and auxin signaling. PLoS Genetics *7*. doi:10.1371/journal.pgen.1002243

Matsuo, N., Makino, M., and Banno, H. (2011). Arabidopsis ENHANCER OF SHOOT REGENERATION (ESR)1 and ESR2 regulate in vitro shoot regeneration and their expressions are differentially regulated. Plant Sci. *181*, 39–46. doi:10.1016/j.plantsci.2011.03.007

Meng, W.J., Cheng, Z.J., Sang, Y.L., Zhang, M.M., Rong, X.F., Wang, Z.W., Tang, Y.Y., and Zhang, X.S. (2017). Type-B ARABIDOPSIS RESPONSE REGULATORs Specify the Shoot Stem Cell Niche by Dual Regulation of WUSCHEL. Plant Cell *29*, 1357–1372. doi:10.1105/tpc.16.00640

Scofield, S., Murison, A., Jones, A., Fozard, J., Aida, M., Band, L.R., Bennett, M., and Murray, J. a. H. (2018). Coordination of meristem and boundary functions by transcription factors in the SHOOT MERISTEMLESS regulatory network. Development. doi:10.1242/dev.157081

Shemer, O., Landau, U., Candela, H., Zemach, A., and Eshed Williams, L. (2015). Competency for shoot regeneration from Arabidopsis root explants is regulated by DNA methylation. Plant Sci. *238*, 251–261. doi: 10.1016/j.plantsci.2015.06.01

Spinelli, S.V., Martin, A.P., Viola, I.L., Gonzalez, D.H., and Palatnik, J.F. (2011). A mechanistic link between STM and CUC1 during Arabidopsis development. Plant Physiol. *156*, 1894–1904. doi:10.1104/pp.111.177709

Takada, S., Hibara, K., Ishida, T., and Tasaka, M. (2001). The CUP-SHAPED COTYLEDON1 gene of Arabidopsis regulates shoot apical meristem formation. Development. *128*, 1127–35.

Tang, X., Bian, S., Tang, M., Lu, Q., Li, S., Liu, X., and Cui, Y. (2012). MicroRNA – Mediated Repression of the Seed Maturation Program during Vegetative Development in Arabidopsis. PLOS Genet. *8*, 20–22.

Wang, J., Tian, C., Zhang, C., Shi, B., Cao, X., Zhang, T.-Q., Zhao, Z., Wang, J.-W., and Jiao, Y. (2017). Cytokinin Signaling Activates WUSCHEL Expression during Axillary Meristem Initiation. Plant Cell *29*, 1373–1387. doi:10.1105/tpc.16.00579

Zhang, T.-Q., Lian, H., Zhou, C.-M., Xu, L., Jiao, Y., and Wang, J.-W. (2017). A Two-Step Model for de novo Activation of WUSCHEL during Plant Shoot Regeneration. Plant Cell. doi:10.1105/tpc.16.00863
